# Dataset for selecting surrogates of emerging biothreat pathogens in laboratory research

**DOI:** 10.1038/s41597-026-07299-9

**Published:** 2026-05-19

**Authors:** Anthony N. Alfredo, Dawn Garrison, Jessica E. Hall, Anna C. Smith, Megan W. Howard, Ryan R. James, Katherine Ratliff, Vipin Rastogi, M. Worth Calfee

**Affiliations:** 1 Battelle Memorial Institute – Columbus, Columbus, OH USA; 2 US Environmental Protection Agency, Research Triangle Park, North Carolina 27711 USA; 3 US Environmental Protection Agency, Ft. Meade, Maryland 20755 USA

**Keywords:** Applied microbiology, Microbiology

## Abstract

Exposure to and transmission of biothreat agents is a serious concern with potentially devastating public health consequences. The rapid development of environmental countermeasure strategies is essential for mitigation efforts, as observed during the recent SARS-CoV-2 pandemic. Only a limited number of laboratories are equipped with the proper containment facilities to handle highly pathogenic biothreat agents. Resources such as Biosafety in Microbiological and Biomedical Laboratories (BMBL), the Bacterial Diversity Database (Bac*Dive*), and the International Committee on Taxonomy of Viruses (ICTV) contain information on bacteria and viruses to aid researchers in selecting non-pathogenic surrogates for laboratory testing aimed at modeling pathogenic agents of interest and developing risk mitigation strategies. Currently, no single, comprehensive dataset exists that includes bacterial and viral surrogate candidates for experimental use. Thus, data from BMBL, Bac*Dive*, ICTV, and other available data sources were retrieved and culled to create the Biothreat Surrogate Alignment (BSA) dataset, a set of pertinent surrogate data. Fields describing possible surrogates that would be most applicable to a specific threat are included.

## Background & Summary

Biological agents released into the environment either by accidental or intentional means can pose significant threats to human health. Following a release incident, the need for rapid and effective environmental countermeasures is of paramount importance. This was most recently made evident during the SARS-CoV-2 Coronavirus Disease 2019 (COVID-19) pandemic, where critical decontamination, sampling, and fate and transport research was limited by availability of the SARS-CoV-2 virus and access to relatively few containment facilities with the capability to work with SARS-CoV-2^1,2^. While increasing the number of available bio-safety level (BSL)-3 laboratories would be capital intensive, valuable testing and research can be performed in lower containment facilities if appropriate non-pathogenic surrogate organisms are identified as suitable representatives for the pathogenic agents of interest. Testing, at least initially, with surrogates offers increased safety, reduced cost, and quantitative efficacy data to support disinfectant selection.

Surrogate microorganisms are employed as a tool to mimic highly pathogenic organisms, and those difficult to acquire or culture. Surrogates are used extensively in agriculture, food safety, aerosol transmission, and disinfection research^1,3–8^. Bacteria and viruses such as *Escherichia coli*, MS2 bacteriophage, *Listeria innocua*, and Murine hepatitis virus are employed as surrogates for pathogens including *Escherichia coli* O157:H7, *Salmonella*, *Listeria monocytogenes*, and SARS-CoV-2^1,2,6,9–11^. Notably, *Bacillus subtilis, B. atrophaeus, and B. thuringiensis* are examples of frequently used surrogates of *B. anthracis*, the causative agent of anthrax, in biothreat research^5,11–15^.

While structural, taxonomic, genomic, and host source information for bacteria and viruses is readily available, determining a proper surrogate for a specific biothreat agent of interest is not trivial. Development of a dataset of surrogate candidates for a biothreat agent based on select characteristics facilitates effectively conducted research studies. Choosing a suitable surrogate depends on several biological and environmental attributes including safety, ease of use, taxonomic similarity, and genetic stability^8^. While others have provided guidance on selection of surrogates based on specific agent characteristics and experimental approach^1,2,7–9,16,17^, no single, comprehensive dataset exists to assist with selection of suitable surrogates for use for emerging pathogen and biothreat agent research. Thus, data from the Bacterial Diversity Database (Bac*Dive*), the International Committee on Taxonomy of Viruses (ICTV), the Biosafety in Microbiological and Biomedical Laboratories (BMBL), and other sources^18^ were retrieved to create the Biothreat Surrogate Alignment (BSA) dataset^19^. The BSA dataset^19^ includes over 12,000 bacteria and 7,000 viruses along with fields of surrogate characteristics such as taxonomic and structural identifiers and BSL that meet customized needs for environmental countermeasure research. The benefits of the BSA dataset^19^ include having characteristics of bacteria and virus in a single dataset. While Bac*Dive* provides extensive search capabilities for bacteria, ICTV allows filtering mostly on taxonomic information, so additional virus information (e.g. ultrastructure, bio-safety level, etc.) included in BSA^19^ provides even more data while maintaining the bacterial information in the same dataset. Limitations of BSA^19^ include some discrepancies between common catalogs, such as American Type Culture Collection (ATCC), due to changes in nomenclature and the large number of strains and sub-species of a given bacteria or virus. The BSA^19^ dataset accomplishes the objective of providing a substantial population of bacteria and virus for surrogate selection.

## Methods

Raw data to be incorporated into the BSA dataset^19^ were compiled primarily from the Bacterial Diversity Database (Bac*Dive*, https://bacdive.dsmz.de) and the International Committee on Taxonomy of Viruses Virus Metadata Resource (ICTV, https://ictv.global/vmr). Bac*Dive* is the largest prokaryote database in the world with data on 97,334 strains^20^. The current release of Bac*Dive* contains information on over 20,000 type strains. The Bac*Dive* website was designed as a comprehensive resource for prokaryote data including taxonomy, physiology, morphology, BSL, and other available information^20^. Similarly, ICTV categorizes viruses through evolutionary relationships^21^. Metadata for viruses within the ICTV database were taken from the Virus Metadata Resource (VMR, https://ictv.global/vmr). Additionally, viral ultrastructure data were sourced from Viral Zone (https://viralzone.expasy.org/). Lastly, BSLs for viruses were determined from Biosafety in Microbiological and Biomedical Laboratories (BMBL), published by CDC and NIH (https://www.cdc.gov/labs/BMBL.html), and includes biosafety principles and criteria (from which acceptable containment levels might be determined).

The ICTV VMR data acquisition was accomplished via a simple file download, but the Bac*Dive* data gathering was more complex. While the Bac*Dive* website offers users the ability to download and filter for specific bacteria, the BSA dataset^19^ includes more bacterial characteristics than are available in the Bac*Dive* filtering tool. To accomplish this, the Bac*Dive* website functionality was used to perform an Advanced Search, filtering for Type Strain = “yes” bacteria. A .csv file with minimal information about each Type Strain = “yes” bacteria was then exported using website functionality. Next, a custom R script was used to leverage the Bac*Dive* application program interface (API) R package to download data of interest for the previously downloaded type strain bacteria. The R script generated a second .csv file export that included fields of interest applicable for filtering potential surrogates.

Logic was used to convert the Bac*Dive* fields into the desired format (e.g., BSL in the Bac*Dive* export can be a list of levels based on different references, in which case the logic selected the highest listed BSL as a “worst case” containment level). In addition, bacteria with records containing no BSL were removed as this was indicative of bacteria that have been the subject of minimal research and thus not likely a suitable surrogate. After data reduction for the current version, 12,173 type strain bacteria remained in BSA^19^.

A similar approach used for bacteria was applied to the virus data obtained from ICTV VMR. VMR data were converted to the desired format using logic (e.g., the genome composition field from VMR is a combination of genome type and single stranded/double stranded data, thus logic was used to populate two fields originating from a single field in ICTV). Viruses that were not of interest were removed from the list based on criteria below:

- VMR Host Source containing “protists”, “archaea”, or indication that the virus was isolated solely from environmental samples. This indicates a virus may be difficult to culture due to host type or that the host is unknown.
- VMR Genome Coverage containing “partial genome”, “no entry in genbank”. If not well documented, the virus is likely understudied and hard to obtain. Limited information about culturing is highly likely.
- Any of the following field values is blank: Phyla, Family, Genus, VMR Virus REFSEQ accession. Incomplete taxonomy likely means the virus is understudied and hard to obtain with limited culturing information available.

After removing viruses that do not apply, 7,083 viruses remained in BSA^19^. The Bac*Dive* data and ICTV VMR data were then merged into a Microsoft Excel spreadsheet.

Finally, SME input was used to fill information gaps within BSA dataset^19^. Input from SMEs included the addition of notes to specific agents and minor formatting and editorial revisions. For example, bacteria and viruses whose possession, use, and transfer are overseen by the US Federal Select Agent Program (referred to as “select agents”) were specified through manual SME inputs. In addition, SME input was used to supplement ICTV viral ultrastructure and BSL information and to specify agents used as EPA regulatory strains.

**Fig. 1:**
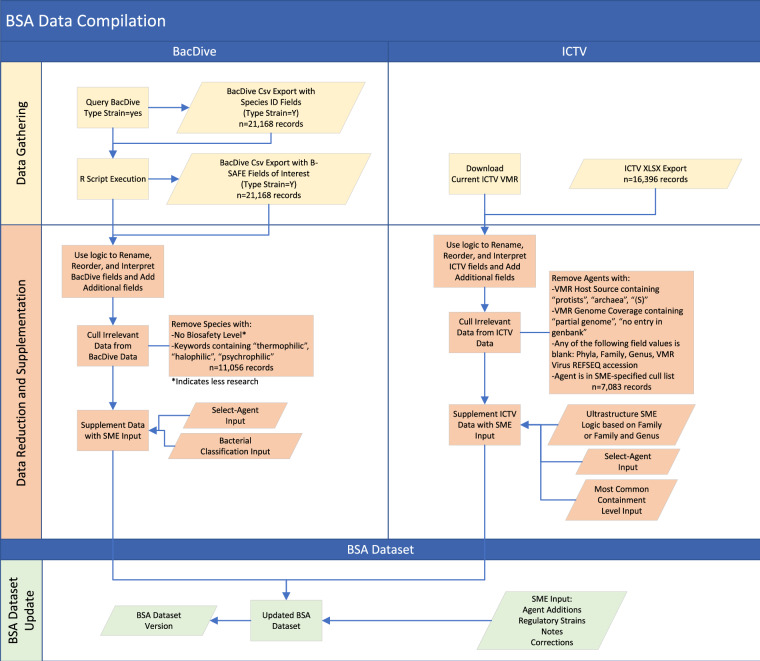
Schematic of the data compilation and supplementation processes of the BSA dataset. Raw data from BacDive and ICTV were exported from their respective websites. Data gathering from both sources filtered information necessary for surrogate selection and was supplemented with subject matter expert (SME) input. Following data recovery, BacDive and ICTV data were merged to generate an updated version of BSA dataset updater.

As discussed above, BSA is a living dataset^19^ comprised of curated Bac*Dive* data, ICTV VMR data, and input from SMEs. Bac*Dive* and ICTV VMR data is continuously refreshed to include research updates concerning bacteria and viruses. Ideally, the BSA dataset^19^ would be based upon the most recent revision of data from those sources. Updating information regularly (perhaps 2-3 times per year) would be beneficial to researchers interested in determining the most suitable surrogates to use for specific experimental studies. An automated BSA dataset^19^ updater was developed to simplify the process of refreshing Bac*Dive* and ICTV VMR data. Figure 1 depicts the complete process for compiling and updating BSA^19^ data.

## Data Records

**Fig. 2:**
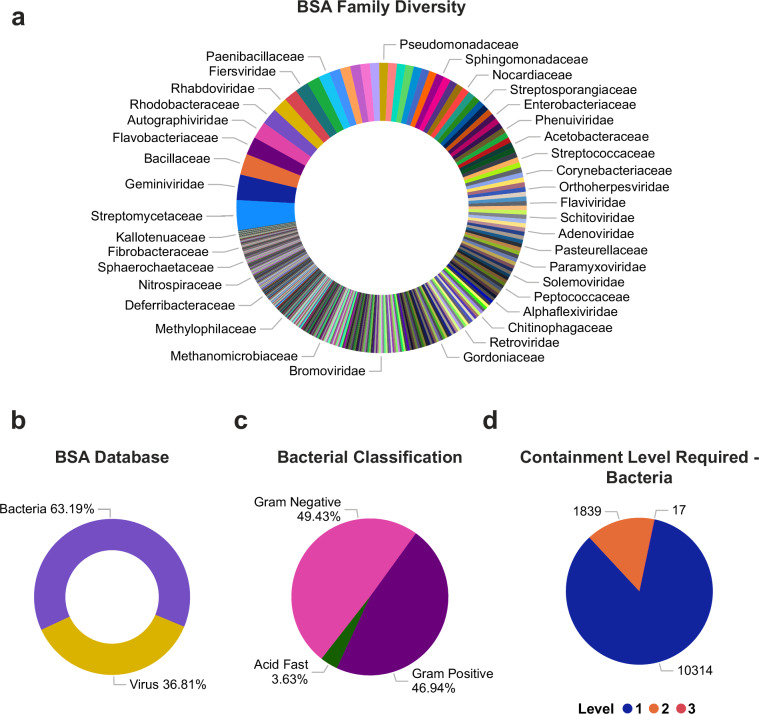
Visual overview of the BSA dataset. (**a**) Family diversity of bacteria and viruses within the BSA dataset. (**b**) The percentage of bacteria (purple) vs. viruses (gold) in the BSA dataset. (**c**) Percentages of different bacteria by cell wall structure. (**d**) Acceptable containment level was determined for database agents based on retrieved data.

In total, 21,168 records from Bac*Dive* and 16,396 ICTV records were imported from the current versions of each data download. Schematic overviews of the contents of the BSA dataset^19^ are shown in Fig. 2 with additional information in Table 1. At the time of publication, BSA^19^ contained 12,173 bacteria and 7,083 viral species with detailed taxonomic, structural, and containment data. Figure 2a show that agents within the BSA dataset^19^ are from over 670 unique taxonomic families, and Fig. 2b–d provide details about the breakdown between bacteria and viruses, bacterial classifications, and containment levels.

**Table 1:** BSA Dataset Overview.

BSA Dataset Content	Yes	No	
Regulatory Agents	28 (0.15%)	19237 (99.85%)	
Aerobic Respiration	5724 (71.44%)	2288(28.56%)	
Spore Forming Agents	1122 (9.43%)	10774 (90.57%)	
Motility	1900 (11.12%)	15189 (88.88%)	
	**DNA**	**RNA**	
Genome	16103 (83.63%)	3151 (16.37%)	
	**Single**	**Double**	
Single / Double Stranded	4204 (21.83%)	15050 (78.17%)	
	**Env**	**Non-env**	**Quasi-env**
Viral Ultrastructure	1689 (25.22%)	5007 (74.76%)	1 (0.02%)

Table 2 provides the fields included in the BSA^19^ dataset that could be used to determine surrogate suitability for bacteria, viruses, or both agent types. Bacteria and viruses were included in BSA^19^ dataset based on criteria provided by either Bac*Dive* or ICTV (e.g., taxonomy, physiology, genome) as well as subject matter expert (SME) input. Most factors were self-explanatory, but there was some nuance to the BSLs included in the dataset. For bacteria, BSLs in BSA^19^ were determined by BSL information from BacDive. Bacteria with unknown containment were listed as 1. For viruses, containment level was determined using BSL information from BMBL or from Risk Group values in NIH Guidelines for Research Involving Recombinant or Synthetic Nucleic Acid Molecules (NIH Guidelines). Like bacteria, viral agents with unknown containment requirements were also considered Level 1. In addition, to avoid producing a dataset encumbered by an excess of organisms that would be difficult to obtain or with little or no information on how to culture or enumerate them, only “type strain” entries from Bac*Dive* were imported. Also, several non-applicable entries such as extremophile bacteria were removed because the environments in which these organisms thrive make them not applicable to environmental countermeasure research. Specifically, Bac*Dive* records with keywords containing “thermophilic”, “halophilic”, or “psychrophilic” were removed. Similarly, for ICTV records, entries with only partial sequences, or viruses only identified in environmental samples with no known host were also removed.

**Table 2:** BSA Dataset Field Descriptions.

Field	Applies To	Description
NCBI tax id	⊠ Bacteria □ Virus	A stable, unique numerical identifier that corresponds to a bacteria node in the taxonomic tree.
Virus GENBANK accession	□ Bacteria ⊠ Virus	A unique, stable alphanumeric identifier assigned to a viral GenBank sequence record.
Kingdom	⊠ Bacteria ⊠ Virus	Bacteria (“B”) or Virus (“V”).
Bacterial Classification	⊠ Bacteria □ Virus	Gram-positive (“G+”), Gram-negative (“G-”) or Acid Fast (“AF”).
Spore	⊠ Bacteria □ Virus	Spore forming (“Y” for yes) or non-spore forming (“N” for no) bacteria.
Acceptable Containment Level	⊠ Bacteria ⊠ Virus	For bacteria, BSL comes from BacDive data. Viral values are assigned based on Biosafety in Microbiological and Biomedical Laboratories (BMBL): https://www.cdc.gov/labs/media/pdfs/2025/08/SF__19a_308133-A_BMBL6_00-BOOK-WEB-final-3.pdf or based on the NIH Guidelines for Research or based on Risk Group values from NIH Guidelines for Research Involving Recombinant or Synthetic Nucleic Acid Molecules (NIH Guidelines) - November 2013. Note the assumption that BSL = Risk Group.
Genome Type	□ Bacteria ⊠ Virus	Viral genome type, either DNA or RNA.
Ultrastructure	□ Bacteria ⊠ Virus	Viral ultrastructure, enveloped (env), non-enveloped (non-env), quasi-enveloped (quasi-env), or not applicable (NA). SMEs used ICTV to inform value assignments to viruses based on phylum or family.
Regulatory Strain / Species	⊠ Bacteria ⊠ Virus	EPA OCSPP 810 guideline regulatory strains (“Y” for yes or “N” for no).
Phyla	⊠ Bacteria ⊠ Virus	Phyla are entered or selected from the dropdown menu.
Order	⊠ Bacteria ⊠ Virus	Order is entered or selected from the dropdown menu.
Family	⊠ Bacteria ⊠ Virus	Family is entered or selected from the dropdown menu.
Genus	⊠ Bacteria ⊠ Virus	Genus is entered or selected from the dropdown menu.
Aerobic	⊠ Bacteria □ Virus	Aerobic or facultative anaerobic (“Y” for yes) or microaerophilic or obligate anaerobic (“N” for no).
Single stranded / Double stranded	□ Bacteria ⊠ Virus	Viral genome material, single stranded (“ss”) or double stranded (“ds”).
Motility	⊠ Bacteria ⊠ Virus	Motile (“Y” for yes) or non-motile (“N” for no) bacteria.
Risk Group	□ Bacteria □ Virus	Values from NIH Guidelines for Research Involving Recombinant or Synthetic Nucleic Acid Molecules (NIH Guidelines) - November 2013.
Select Agent	⊠ Bacteria ⊠ Virus	Based on Select Agents and Toxins List https://selectagents.gov/ from May 29, 2024. (“Y” for yes or “N” for no).
Notes	⊠ ⊠ Bacteria ⊠ Virus	Mainly used to explain BSL value assignments made. Can be used to detail other value assignments.
Source	⊠ Bacteria ⊠ Virus	Bacteria from BacDive: https://bacdive.dsmz.de/advsearch Viruses from ICTV: https://ictv.global/sites/default/files/VMR/VMR_MSL39.v1_20240912.xlsx SME Additions: blank

## Technical Validation

To validate the data used to build the BSA dataset^19^, we developed an Excel-based validation tool for the BSA and compared the contents of the dataset to the ATCC database (https://www.atcc.org/). Search queries were evaluated to determine if provided organisms could be readily acquired through ATCC. To accomplish this, the BSA validation tool was queried for potential surrogates of bacterial and viral agents listed by the US Federal Select Agent Program. These biological agents have been identified as potential threats to human and/or animal health by the Centers for Disease Control and Prevention (CDC) and the United States Department of Agriculture’s (USDA) Animal and Plant Health Inspection Service (APHIS) and represent over 15 unique genera. Select agents were used for technical evaluation of BSA validation tool queries via genus searches, and the number of returns was noted. For a select agent genus with few results, searches were modified by using the family of these agents.

**Fig. 3:**
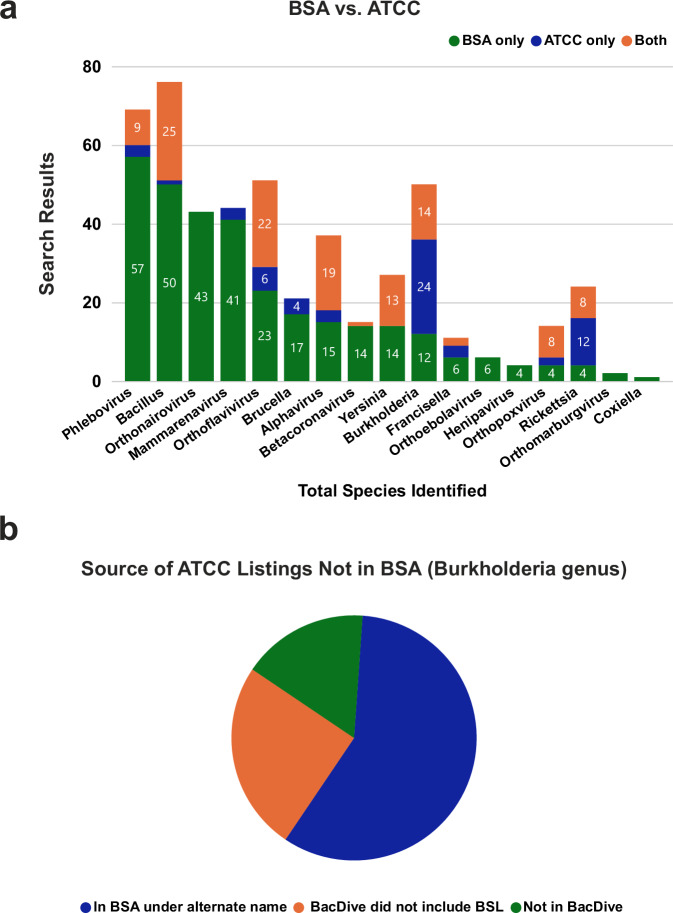
Technical validation of BSA dataset where genus search queries for select agents (defined by the US Federal Select Agent Program) were searched in both BSA and ATCC engines. (**a**) Results of BSA dataset searches were compared to ATCC. (**b**) Breakdown of Burkholderia genus search returns not identified in the BSA dataset.

For each BSA^19^ search query, the genus of a particular select agent was used to determine genetically similar organisms to be used for environmental countermeasure research. The results with 100% matching criteria provided by the validation tool were compared to results from the ATCC database for the same genus. The results of the technical evaluation of BSA^19^ are illustrated in Fig. 3, where Fig. 3a shows a graphical representation of each select agent genus and the total number of search returns per genus identified by BSA only (green), ATCC only (blue), or both (orange). This demonstrates the capability of BSA^19^ data to be used to identify commercially available agents as potential surrogates. For example, when searching the BSA^19^ dataset and ATCC for the genus *Bacillus*, the results returned 50 species in BSA^19^ and 25 species that were available in both the BSA dataset^19^ and ATCC. During this evaluation, it was also noted that24 bacteria of genus *Burkholderia* were found in ATCC only and not the BSA dataset^19^. Of those queried, this was the largest discrepancy between ATCC and the BSA dataset^19^, so further investigation was performed to understand the root cause. As shown in Fig. 3b, of the 24 *Burkholderia* bacteria names only in the BSA dataset^19^, it was determined that 14 (58%%) were in BSA^19^ but listed under a different name (e.g., *Burkholderia fungorum* in ATCC was listed as *Paraburkholderia fungorum* in BSA^19^) and of the remaining 10 organisms, six of the ATCC listings missing from BSA^19^ were initially filtered from dataset inclusion because Bac*Dive* did not include a BSL (a BSL is required for BSA^19^,as missing BSLs indicate a lack of organism research) for those agents. The final four missing bacteria are not in Bac*Dive* and thus were not imported into the BSA dataset^19^.

This example demonstrates some of the limitations of the BSA^19^ dataset including discrepancies between other databases (e.g. ATCC, Bac*Dive*) due to changes or differences in nomenclature, the large number of strains and sub-species of a given bacteria or virus for a given species in ATCC (BSA^19^ was not designed for strain level surrogate identification), and the updating process of the dataset. The objective of BSA^19^ was not to catalog all the bacteria and viruses available but rather contain a substantial population of choices to facilitate surrogate selection, with many of the non-ideal surrogate candidates removed by logic (rather than individually) to make the dataset more user-friendly.

## Usage Notes

The BSA^19^ dataset was designed to provide a dataset of possible surrogates for microbes of interest. In addition to the dataset made available in the repository, the query tool referenced in the Technical Validation section (Biothreat Surrogate Alignment and Filtering Engine, B-SAFE) is available via the References download section at 10.23719/1532283, or directly downloaded at https://pasteur.epa.gov/uploads/10.23719/1532283/documents/B-SAFE%20Tool%20version%205-0.xlsx. Users may query the BSA^19^ dataset based on the available fields to return a list of potential surrogates to facilitate environmental countermeasure and decontamination research pertaining to emerging pathogens and future pandemic threats.

## Data Availability

The BSA dataset is available on data.gov at 10.23719/1532283, via the Downloads and Resources section. The B-SAFE query tool is also available at 10.23719/1532283, via the References section, or directly downloaded at https://pasteur.epa.gov/uploads/10.23719/1532283/documents/B-SAFE%20Tool%20version%205-0.xlsx. R script was used to efficiently obtain data of interest from the BacDive repository and is available at https://github.com/calfee-worth/B-SAFE.git.
